# Electronic medical record-based causal network modeling for acute myocardial infarction diagnosis in the emergency department

**DOI:** 10.1016/j.isci.2026.115742

**Published:** 2026-04-16

**Authors:** Bo-Yuan Li, Xue-Qi Li, Yu-Tong Jiang, Xiao-Yang Li, Zhao-Xing Tian, Rui Kang

**Affiliations:** 1 School of Reliability and Systems Engineering, Beihang University, Beijing 100191, China; 2 Department of Emergency Medicine, Beijing Jishuitan Hospital, Capital Medical University, Beijing 100035, China

**Keywords:** Health sciences, Medicine, Emergency medicine, Health informatics, Cardiovascular medicine

## Abstract

For the acute myocardial infarction (AMI) diagnosis in the emergency department, the atypical manifestations and limited information lead to clinical challenge. Data-driven methods often fail in the generalizability against the atypical and limited information. In this work, the causality of AMI is studied based on electronic medical record (EMR), and a framework to construct causal network for AMI diagnosis is proposed. The EMRs with seven categories and 6,001 samples are included. Score-based algorithm, structural equation model, and network coarse-graining are adopted to build causal network with medical knowledge. A model validation procedure is proposed to test the model performance when only part of variable information is obtained. Compared with data-driven methods, causal network achieves best comprehensive performance. Further, the causal effects between variables and AMI can be quantified, which are verified by the sensitivity analysis on unobserved confounders. Such results can support the disease diagnosis, treatment, and healthcare in clinic.

## Highlights

• The causal network for AMI diagnosis is built with real EMR data in the emergency
• Network coarse-graining method is proposed for eliminate undirected edges
• The model performance is validated when only part of information can be obtained
• The causal effects between variables and the onset of AMI are quantified

## Graphical abstract

**Figure:**
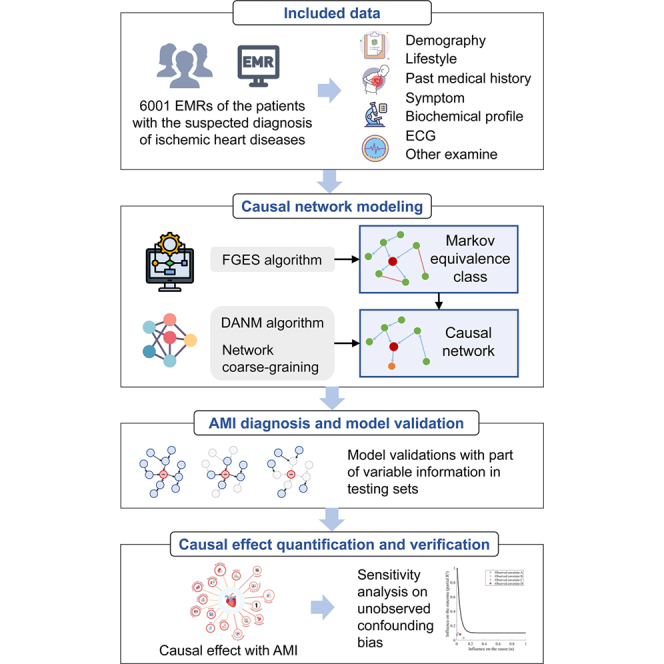

## Introduction

Acute myocardial infarction (AMI) is defined pathologically as myocardial cell death due to prolonged ischemia. The abnormal changes in cells occur as early as 10–15 min after coronary occlusion, and necrosis can progress from the subendocardium to the subepicardium over several hours.^1^ Since AMI develops rapidly, urgent evaluation and medical strategy with time measured by minutes are required in clinic.^2^ In addition, AMI is with high mortality: it causes more than 2.4 million deaths in the USA, more than 4 million deaths in Europe and northern Asia, and more than a third of deaths in developed nations annually.^3^

Generally, typical manifestations of electrocardiogram (ECG) ST segment (the interval from the end of the S wave to the begining of the T wave in a ECG) and cardiac troponin (cTn) are regarded as critical variables for AMI diagnosis. However, in the emergency department, a considerable proportion of patients may not have typical manifestations, leading to misdiagnosis. Epidemiological studies indicated that 26% of AMI patients can present without typical chest pain^4^; meanwhile, the cases of AMI patients with atypical symptoms such as dizziness have been reported and reviewed.^5^ In particular, an observational study^6^ in a UK General Hospital emergency department found that 63.04% (58/92) of patients with severe coronary artery stenosis did not follow the standard 4th universal definition of AMI, which shows the limited discriminatory value of typical manifestations in patients with potential AMI. To accurately diagnosis AMI considering the aforementioned atypical manifestations, it is important to comprehensively evaluate AMI risk with large-scale and multi-category variables. However, considering the time constraint in the emergency department, it is difficult to obtain sufficient variable information in time. This leads to data missing for the AMI diagnosis in clinic, and it also suggests that a simplified priority checklist in the emergency department is critical for rapid and accurate decisions.

Nowadays, with the improvement of medical datasets such as electronic medical record (EMR), the data-driven algorithms were utilized to find the correlations between cardiovascular events and diverse variables,^7^^,^^8^^,^^9^ which are expected to support AMI diagnosis. However, the data-driven methods study only correlations without considering causality. Essentially, these methods fit the distributional features in the training data but not discuss the deterministic mechanisms between variables. Therefore, when the correlation-based models are transferred to the testing data that are significantly different from the training set (e.g., the atypical manifestations and data missing in the emergency department), the statistical correlations built into the models may be invalidated. Consequently, the prediction performance declines, resulting in poor generalizability.^10^ In addition, although such methods can identify feature contributions to AMI diagnosis, they cannot effectively distinguish the causality and the association due to confounders. Therefore, it may be difficult for data-driven methods to clarify intervention outcomes and guide disease treatment and healthcare.^11^

To solve this problem, the causality related to the onset of AMI should be studied to improve the generalization and explainability.^12^ The essence of causality is to determine whether a correlation of two variables is due to their direct link, other mediate variables, or confounders. In other words, causality studies the fundamental sources of variable correlations. The variations and noise in data may disturb variable correlations but is difficult to disturb the sources behind correlations. Therefore, causal methods are more robust compared with the data-driven methods only focusing on correlations but not their sources.

For this point, epidemiological studies generally explore causalities based on the methods such as Mendelian randomization to exclude the effects of confounders, and the sensitivity analysis can be performed to ensure the robustness of causalities. In addition, the typical causal method, Bayesian network has been applied in the prediction of cardiovascular diseases. Fuster-Parra et al. built the Bayesian network of cardiovascular risk based on workers’ annual health assessment data, which contained 13 variables such as age, sex, and lipid^13^; Orphanou et al. studied the 1,417 patients’ data, and adopted the dynamic Bayesian network to establish the temporal causalities for coronary heart disease and 18 variables such as diabetes and obesity^14^; Tylman et al. constructed the Bayesian network expected to support the AMI diagnosis in the emergency department, which contained 19 variables including ECG features and biochemical profiles.^15^ But its parameters were determined by literature and expert knowledge, and only 29 patient cases were used for validation.

For such studies, many variables that have significant relationships with cardiovascular diseases have not been considered yet, such as: symptoms like chest pain, and past medical history including cerebrovascular disease (CVD) and chronic renal insufficiency. This may be because it is difficult to collect and process the numerous real EMR data with various categories, and extract useful information from it.^16^ Studying the causalities related to the above-mentioned variables not only ensures the diagnostic accuracy but also provides important information to explore the pathophysiological mechanisms of AMI. Therefore, the causal network modeling with more categories and quantities of variables is valuable.

Based on Bayesian network, structural causal model (SCM) has been developed, which can construct causal network integrating statistical data and prior knowledge.^17^ By automatic construction, SCM can discover the causality among large-scale variables; it may also find some new causalities that have not been considered. In addition, by introducing the concept of intervention, the causal effects between variables can be measured in the causal network. The comparison between traditional Bayesian network and causal network is concluded as shown in Table 1.

**Table 1:** Comparison between Bayesian network and causal network

Factor	Bayesian network	Causal network
Construction	manually	automatically
Included variables	less	more
Constructed causalities	known	possibly new
Causal effect quantification	no	yes

Based on the method of SCM, in this work, more variables in the real EMR data are considered. In particular, we collected and studied the real EMR data of 6,001 patients from the emergency department of a hospital in Beijing, China. Considering the clinical relevance and data availability in the emergency department, seven categories of variables are collected for each patient: demography, lifestyle, past medical history, symptom, biochemical profile, ECG, and other examine, with a total of 54 variables.

When discovering causalities among numerous variables, it can be challenging to identify causal directions, which may be caused by bidirectional causalities or unobserved confounders. Therefore, the solution of the causal discovery from limited observational data is usually non-unique,^18^^,^^19^ bringing challenges to model the causal network for AMI diagnosis.

To address this issue, in this work, the score-based algorithm is adopted to construct the Markov equivalence class (MEC), and the unique solution of the causal network can be determined by combining structural equation model (SEM) and network coarse-graining. Based on the established causal network, the probability of AMI onset can be inferred to achieve AMI diagnosis. Further, the diagnostic performance of causal network is compared with those of data-driven methods, particularly under the condition that only part of variable information can be obtained, corresponding to the data missing in the emergency department. Finally, the causal effects among variables can be quantified by effective information (EI), which can support the treatment and health management of AMI.

## Results

### Study population and included variables

**Figure 1:**
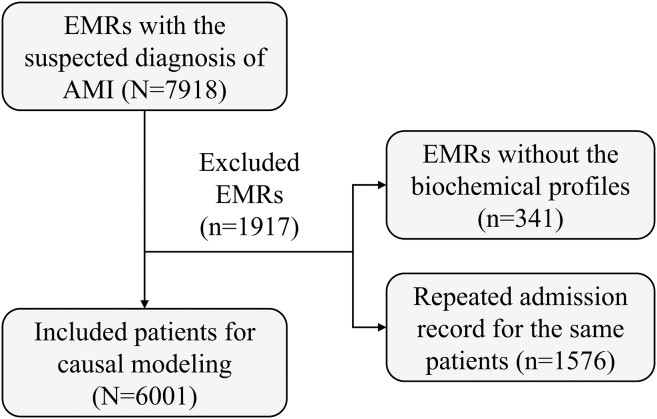
The flowchart to include the studied cohort EMR, electronic medical record; AMI, acute myocardial infarction.

A total of 7,918 EMRs in the emergency department of Beijing Jishuitan Hospital (Beijing, China) are collected in this work, which were all with the suspected diagnosis of ischemic heart diseases. Based on the inclusion and exclusion criteria as shown in Figure 1, 6,001 patients are included in this work, in which 2,731 patients were finally diagnosed with AMI.

For each patient, the variables included in this work should associate with the onset of AMI, and be available in the EMRs of emergency department. When including variables, clinical knowledge including the widely accepted guidelines for the management of acute coronary syndromes (ACSs) is referred.^20^ Also, the included variables are discussed by an experienced clinical team. Finally, seven categories with a total of 54 variables are included: demography, lifestyle, past medical history, symptom, biochemical profile, ECG, and other examine.

Considering clinical practical significance, these variables are discretized. Since the pathological physiological mechanisms are complex and lacking cognition, it is difficult to clarify the precise relationship between continuous changes in variables and disease states. Take cTn for instance: for an individual patient, it is difficult to assert that a cTn level of 10 represents severe ischemic condition than a level of 9.5 (unit: pg/mL). Actually, the binarized or ternarized discretization of biochemical variables is a widely accepted practice in clinical diagnosis, especially in the emergency department. To guide in-time triage, it is important to determine whether a biochemical variable is pathological or not, but not precisely study its numerical value. Clinicians routinely use the threshold-based discretization of biochemical variables to judge the patients’ states, as recommended in clinical guidelines.^20^

In this work, the discretization thresholds are also set according to clinical guidelines, including European Society of Cardiology (ESC) guidelines for the management of ACSs, American Heart Association (AHA)/American College of Cardiology (ACC) guideline on the management of blood cholesterol, American Association for Respiratory Care (AARC) clinical practice guideline, European Society for Clinical Nutrition and Metabolism (ESPEN) practical guideline, etc. Also, discretization thresholds are discussed by clinical team to ensure the rationality. Also take cTnI for instance: according to the 4th universal definition,^1^ the cTnI level above the healthy population-defined 99th percentile is regarded as an abnormal elevation. Since the 99th percentiles are diversely valued in different assay specifications, the threshold of cTnI is determined by the assay specifications of the analyzers adopted in Beijing Jishuitan Hospital. The discretization is important to ensure the model interpretability: clinicians in the emergency department can quickly understand the model based on the knowledge consistent with guidelines, without having to interpret the complex mappings between continuous variables.

Conclusively, statistics of the included variables can be referred to in Table 2.

**Table 2:** Statistics of the included variables

Category	Variable	Discretization criteria	AMI = 0 (3,270)	AMI = 1 (2,731)	***χ***^2^	*p* value
Demography	age ( per year)	0: ≤30	30	2	228.885	<0.0001
		1: 31–50	181	259		
		2: 51–70	1,404	1,576		
		3: >70	1,655	894		
	sex	0: female	1,568	1,020	68.199	<0.0001
		1: male	1,702	1,711		
Lifestyle	smoking history (smoking)	0: no	2,457	1,574	206.7481	<0.0001
		1: yes	813	1,157		
	drinking history (alcohol)	0: no	2,706	1,958	105.069	<0.0001
		1: yes	564	773		
Past medical history	cardiovascular disease (CAD)	0: no	977	502	105.900	<0.0001
		1: yes	2,293	2,229		
	cardiac dysfunction (CD)	0: no	2,642	1,294	736.129	<0.0001
		1: yes	628	1,437		
	hypertension (HTN)	0: no	1,149	816	18.685	<0.0001
		1: yes	2,121	1,915		
	diabetes	0: no	1,966	1,613	0.694	0.4047
		1: yes	1,304	1,118		
	dyslipidemia (DYS)	0: no	1,965	563	951.241	<0.0001
		1: yes	1,305	2,168		
	chronic renal insufficiency (CRI)	0: no;	2,989	2,527	2.528	0.1118
		1: yes	281	204		
	thyroid disease (TD)	0: no	2,579	2,103	3.014	0.08253
		1: yes	691	628		
	Percutaneous coronary intervention history (PCI)	0: no	2,620	1,445	504.227	<0.0001
		1: yes	650	1,286		
	valvular heart disease (VHD)	0: no	3,179	2,587	24.520	<0.0001
		1: yes	91	144		
	cerebrovascular disease (CVD)	0: no	2,538	2,467	173.902	<0.0001
		1: yes	732	264		
	nutrition	0: no	2,993	2,701	166.633	<0.0001
		1: yes	277	30		
Symptom	chest pain	0: no	2,924	1,371	1,124.896	<0.0001
		1: yes	346	1,360		
	arhythmia (A-ryth)	0: no	3,247	2,692	7.643	0.005698
		1: yes	23	39		
	cardiogenic shock (CS)	0: no	3,244	2,695	3.982	0.04598
		1: yes	26	36		
	limb numbness (numbness)	0: no	2,785	2,626	202.641	<0.0001
		1: yes	485	105		
	pulmonary rales (PR)	0: no	3,072	2,633	19.309	<0.0001
		1: yes	198	98		
	dyspnea	0: no	2,924	1,940	327.460	<0.0001
		1: yes	346	791		
Biochemical profile	uric acid (UA) (μmol/L)	0: <90	9	2	15.115	0.0005221
		1: 90–420	2,910	2,356		
		2: >420	351	373		
	creatinine (Cr) (μmol/L)	0: <53	381	230	27.521	<0.0001
		1: 53–140	2,747	2,422		
		2: >140	142	79		
	triglyceride (TG) (mmol/L)	0: <0.45	19	6	32.087	<0.0001
		1: 0.45–1.69	2,813	2,223		
		2: >1.69	438	502		
	total cholesterol (TC) (mmol/L)	0: <2.86	266	224	0.167	0.9197
		1: 2.86–5.98	2,911	2,434		
		2: >5.98	93	73		
	albumin (ALB) (g/L)	0: <35	391	145	84.211	<0.0001
		1: 35–50	2,879	2,583		
		2: >50	0	3		
	urea (mmol/L)	0: <2.5	22	8	22.731	<0.0001
		1:2.5–7.5	2,802	2,448		
		2: >7.5	446	275		
	erythrocyte sedimentation rate (ESR) (mm/h)	0: ≤20	2,892	2,532	31.264	<0.0001
		1: >20	378	199		
	hemoglobin (HGB) (g/L)	0: <110	944	286	323.204	<0.0001
		1: 110–160	2,254	2,316		
		2:>160	72	129		
	white blood cell (WBC) (10^9^/L)	0: <4	167	109	89.523	<0.0001
		1: 4–10	2,464	2,320		
		2: >10	639	302		
	lymphocyte count (LYM#) (10^9^/L)	0: <0.8	471	156	122.997	<0.0001
		1: 0.8–3.5	2,770	2,535		
		2: >3.5	29	40		
	neutrophil (NEUT) (%)	0: <50	173	197	233.811	<0.0001
		1: 50–70	1,630	1,829		
		2: >70	1,467	705		
	platelet (PLT) (10^9^/L)	0: <150	564	327	37.163	<0.0001
		1: 150–450	2,677	2,391		
		2: >450	29	13		
	homocysteine (Hcy) (μmol/L)	0: ≤15	2,706	2,160	13.001	0.0003113
		1: >15	564	571		
	potassium (K) (mmol/L)	0: <3.5	270	197	3.386	0.1840
		1: 3.5–5.5	2,985	2,526		
		2: >5.5	15	8		
	sodium (Na) (mmol/L)	0: <136	180	51	53.342	<0.0001
		1: 136–146	2,993	2,591		
		2: >146	97	89		
	total bilirubin (TBIL) (μmol/L)	0: <3.42	1	0	2.504	0.2860
		1: 3.42–20	2,925	2,415		
		2: >20	344	316		
	cardiac troponin I (cTnI) (pg/mL)	0: ≤17.5	3,047	2,688	96.645	<0.0001
		1: >17.5	223	43		
	creatine kinase isoenzyme (CK-MB) (ng/mL)	0: <0.6	28	17	17.527	0.0001563
		1: 0.6–6.3	3,142	2,574		
		2: >6.3	100	140		
	D-dimer (mg/L)	0: ≤0.5	2,342	2,370	202.800	<0.0001
		1: >0.5	928	361		
	thrombin time (TT) (s)	0: <9	0	1	1.440	0.4868
		1: 9–19	3,167	2,650		
		2: >19	103	80		
	prothrombin INR (INR)	0: <0.9	51	36	76.274	<0.0001
		1: 0.9–1.1	2,841	2,552		
		2: >1.1	378	143		
	fibrinogen (FIB) (mg/dL)	0: <200	65	37	92.132	<0.0001
		1: 200–400	2,741	2,511		
		2: >400	464	183		
	PH	0: <7.35	76	51	8.906	0.01164
		1: 7.35–7.45	3,067	2,608		
		2: >7.45	127	72		
	partial pressure of oxygen (PO2) (mmHg)	0: <80	393	183	66.802	<0.0001
		1: 80–110	2,724	2,472		
		2: >110	153	76		
	partial pressure of carbon dioxide (PCO2) (mmHg)	0: <35	469	238	57.015	<0.0001
		1: 35–45	2,738	2,467		
		2: >45	63	26		
	lactic acid (Lac) (mmol/L)	0: <0.5	12	4	9.378	0.009196
		1: 0.5–1.6	2,968	2,534		
		2: >1.6	290	193		
ECG	ST segment abnormality (STSA)	0: no	2,968	1,898	438.838	<0.0001
		1: yes	302	833		
	T wave abnormality (TWA)	0: no	3,207	2,488	149.451	<0.0001
		1: yes	63	243		
	R wave abnormality (RWA)	0: no	3,266	2,714	10.675	0.001086
		1: yes	4	17		
Other examine	left ventricular ejection	0: <50%	19	27	3.250	0.07140
		1: ≥50%	3,251	2,704		
	systolic blood pressure (SBP) (mmHg)	0: <90	38	57	14.262	0.0008001
		1: 90–140	2,010	1,746		
		2: >140	1,222	928		
	pulse (beats/min)	0: <60	184	258	45.748	<0.0001
		1: 60–100	2,821	2,326		
		2: >100	265	147		
	respiratory rhythm (ResRhy) (breaths/min)	0: <10	2	1	27.829	<0.0001
		1: 10–24	3,213	2,722		
		2: >24	55	8		

### Causal network modeling

**Figure 2:**
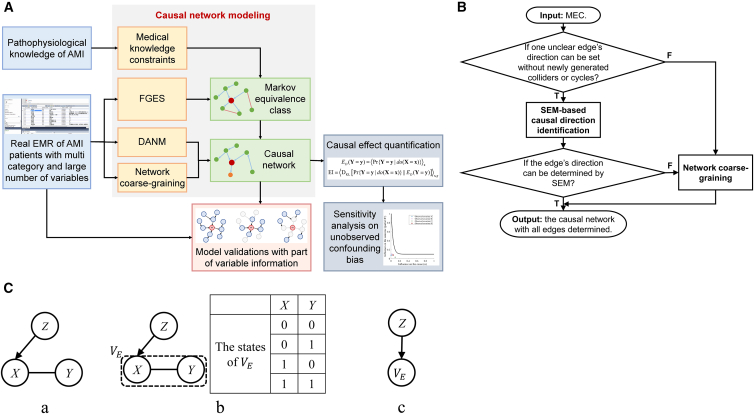
Framework of the proposed method and detailed procedure of causal network identification (A) The framework of the proposed method, which can be mainly divided as causal network modeling, model validation, causal effect quantification, and sensitivity analysis on unobserved confounding bias. EMR, electronic medical record; AMI, acute myocardial infarction; FGES, fast greedy equivalence search; DANM, discrete nonlinear additive noise model. (B) The procedure for causal network identification with structural equation model (SEM) and network coarse-graining. Under the premise that no new V-structure or cycle generates, SEM is adopted to identify directions. If the direction cannot be identified or there must be new V-structure or cycle, network coarse-graining is performed to cover the unclear edges. MEC, Markov equivalence class. (C) An instance of network coarse-graining, in which: (a) *Y* to *X* introduces a new V-structure, and assume that structural equation model (SEM) cannot identify *X* to *Y*; (b) Integrate *X* and *Y* as a macro variable, *V*_*E*_. The states of *V*_*E*_ are the combinations of the states of *X* and *Y*. (c) Rebuild the causality between *Z* and *V*_*E*_.

When constructing the causal network, the medical knowledge should be considered as constraints to ensure the rationality of causal modeling. On this basis, a score-based causal discovery algorithm, the fast greedy equivalence search (FGES), is adopted to construct the MEC, which can effectively deal with the numerous variables related to AMI.^21^ To obtain the unique causal network from the MEC, the causal directions can be identified by a typical SEM, discrete nonlinear additive noise model (DANM), without introducing new V-structures (the three-node structure that *A*→*B* and *C*→*B* with *A* and *C* being non-adjacent) or cycles; otherwise, the network can be locally coarse-grained to cover the uncertain causalities. The framework of causal modeling can be referred to in Figure 2A. For detailed methodology, refer to STAR Methods. Also, Figure 2B shows the procedure for causal network identification with SEM and network coarse-graining, and Figure 2C illustrates an instance of network coarse-graining.

**Figure 3:**
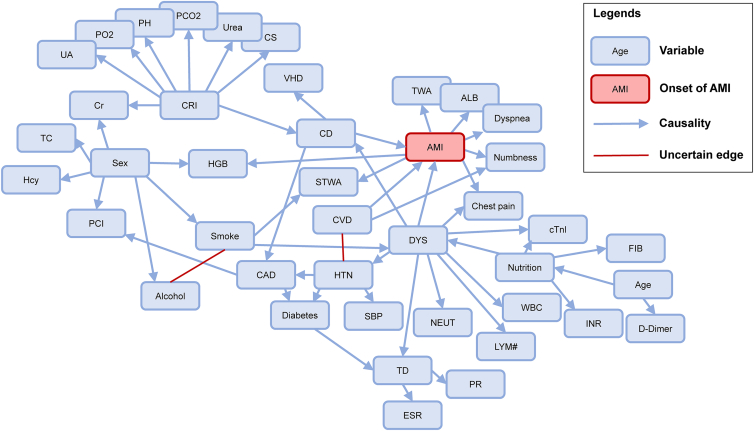
MEC for the onset of AMI in the emergency department Each node represents a variable. Arrows between nodes are the discovered causalities, and the red edges are the unclear associations. MEC, Markov equivalence class; AMI, acute myocardial infarction. Full name of variables in the network can be referred to in Table 2.

Based on the collected EMRs, the constructed MEC are illustrated in Figure 3. It contains 42 variables and 54 causal edges (for details, see Table S1), and there are two unclear directions: the one between smoking (representing smoking history) and alcohol (representing drinking history), and the one between HTN (hypertension) and CVD.

For the edge between HTN and CVD, the direction of HTN → CVD cannot introduce new V-structures or cycles, while CVD → HTN can introduce V-structures (i.e., CVD → HTN ← DYS). Therefore, DANM is adopted to test the direction of HTN → CVD. The residual error of discrete regression is independent with HTN when fitting the function from HTN to CVD (the *p* value of Pearson’s chi-square test is 0.2803 > 0.05, suggesting no significant correlation), which means the causality from HTN to CVD can characterize the unclear association between these two variables.

However, for the edge between smoking and alcohol, although both directions do not introduce V-structures due to their links with sex, DANM also cannot identify a clear direction with independent residual error. It may suggest that there is a coupling bidirectional mechanism. Some studies also have proved that from the perspectives of physiology and psychology, smoking and drinking may have complex synergy and mutual promotion.^22^ In this case, the independent effects of these two variables on AMI cannot be distinguished. Using the network coarse-graining, smoking and alcohol are integrated as one macro variable called lifestyle. It can be referred as a four-valued variable: neither smoke nor drink; only smoke; only drink; smoke and drink. Since the former MEC contains the causalities from sex to smoking and alcohol, and the ones from smoking to DYS (dyslipidemia) and STSA (ST segment abnormality), lifestyle also can be regarded to have these causalities, that is, sex influences lifestyle, and lifestyle affects DYS and STSA. These edges are reconstructed in the MEC to obtain the final causal network after coarse-graining.

**Figure 4:**
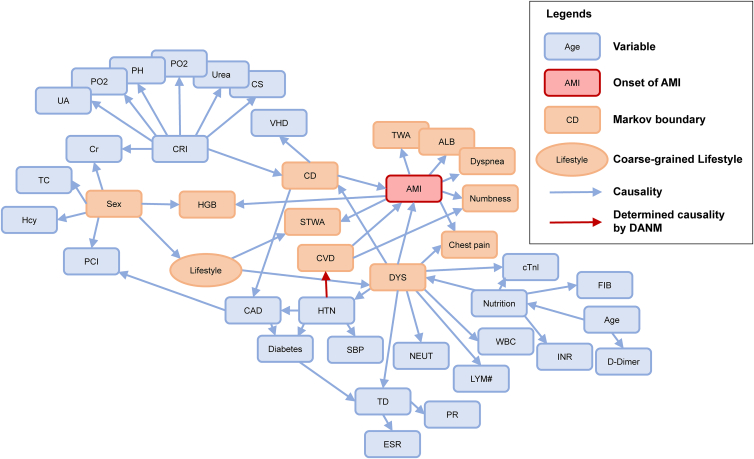
Causal network for the onset of AMI in the emergency department The variables in the Markov boundary of acute myocardial infarction (AMI) are highlighted, as well as the coarse-grained macro variable lifestyle. The causality identified by the discrete nonlinear additive noise model (DANM) is remarked. Full name of variables in the network can be referred to in Table 2.

Finally, the established causal network is presented in Figure 4. In particular, the Markov boundary for the onset of AMI can be identified. The Markov boundary of AMI is the variable set composed of its parent variables, its children variables, and its children’s other parent variables. This is the minimal variable set excluding AMI, conditional on which AMI is independent of other variables in the network. Therefore, it is enough to infer the distribution of AMI given its Markov boundary, without the need for other variables.

As highlighted in Figure 4, the Markov boundary of AMI includes 12 variables: sex, lifestyle, DYS, CVD, CD (cardiac dysfunction), chest pain, numbness, dyspnea, ALB (albumin), HGB (hemoglobin), STSA, and TWA (T wave abnormality). From the perspective of causal network, these variables are the necessary variables to ensure the optimal diagnostic performance. Meanwhile, these variables are generally focused by clinicians in the emergency department. In particular, chest pain and typical ischemic ECG features (STSA and TWA) are critical indicators to define AMI according to the 4th universal definition.^1^ Sex, DYS, CD, CVD, and lifestyle (smoking and alcohol) are also regarded as high-risk factors of AMI, which have been recorded in 2023 ESC guidelines for the management of ACS.^20^ In addition, the atypical symptoms of numbness and dyspnea,^23^^,^^24^ and the changes in ALB^25^ and HGB^26^^,^^27^ have been found after the onset of AMI. Therefore, the critical variable set of Markov boundary is generally aligned with the cognition in clinical practice.

For the established causal network, since the studied data are from the real EMRs of emergency department, two remarks should be further discussed.

(1) The EMRs reflect the cross-sections that the patients visited the emergency departments. With the EMRs, the causalities of biomarkers such as cTn may not be significant. It may be due to the incomplete necrosis of myocardial cells, the delayed rise of cTn, or other individual factors. Detailed analysis on this issue is provided in discussion.
(2) Since the EMRs are recorded for the patients in the emergency department, the distributions of the variables’ values may be different from those of healthy population. For instance, 75.35% of the included patients had a history of cardiovascular disease. Therefore, the established model should differentiate AMI from other potential diseases.

The above characteristics are exactly the typical features of patients in the emergency department. Therefore, the applicable scenario of the established causal network should be strengthened. It is expected to be applied for the AMI diagnosis in the emergency department. When applied to other scenarios such as daily monitoring, the causal network should be updated based on the corresponding data.

### AMI diagnosis and model validation

Based on the established causal network, the network parameters are trained with Bayesian estimation method, and the probability distribution of AMI can be inferred. Although diagnostic models are trained using all 54 variables. However, in the emergency department, the variables serving as the input of diagnostic models may be limited; due to that, some detections and examinations cannot be performed in time. This leads to the data missing in testing sets when verifying the diagnostic performance.

**Figure 5:**
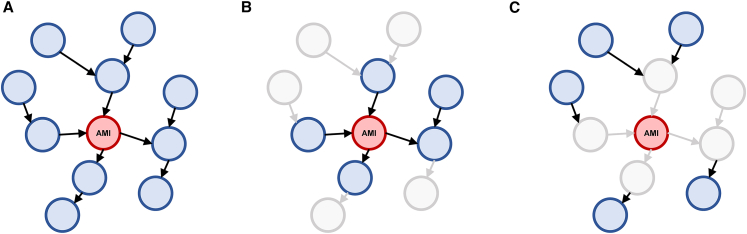
Schematic of the variables included in the testing set for each validation (A) The testing set includes all variables except the onset of acute myocardial infarction (AMI). (B) The testing set only includes the Markov boundary of AMI. (C) The testing set only includes the complementary set of the Markov boundary, also excluding AMI.

Therefore, three validations are performed (respectively referred as validation A, B, and C), in which the training sets include all variable information, but the testing sets could contain only part of variable information. The variables in each testing set can be referred to in Figure 5. Further, to verify the necessity to consider the causality, three prominent data-driven algorithms, XGBoost,^28^ LightGBM,^29^ and Tabnet^30^ are adopted for comparison.

For validation A, the testing sets include all variables excluding the onset of AMI. The performance comparison is depicted in Table 3.

**Table 3:** Performance comparison for validation A

Method	Accuracy	Sensitivity	Specificity	Precision	F1-score	Log loss
Causal network	0.794 ± 0.0190	0.746 ± 0.0271	**0.835** ± **0.0165**	**0.790** ± **0.0252**	0.767 ± 0.0214	0.496 ± 0.0406
XGBoost	0.808 ± 0.0172	0.794 ± 0.0218	0.820 ± 0.0216	0.787 ± 0.0233	0.790 ± 0.0185	0.457 ± 0.0242
LightGBM	**0.816** ± **0.0208**	**0.815** ± **0.0193**	0.817 ± 0.0271	0.789 ± 0.0274	**0.802** ± **0.0212**	**0.424** ± **0.0188**
Tabnet	0.802 ± 0.0166	0.782 ± 0.0301	0.819 ± 0.0264	0.784 ± 0.0237	0.783 ± 0.0188	0.452 ± 0.0234

Bold indicates the method with the best performance among all methods.

Result suggests that the performance of the proposed model is similar with those of data-driven methods, given all variable information for testing sets. For the validation B using testing sets with the Markov boundary, its result is depicted in Table 4.

**Table 4:** Performance comparison for validation B

Method	Accuracy	Sensitivity	Specificity	Precision	F1-score	Log loss
Causal network	**0.794** ± **0.0190**	**0.746** ± **0.0271**	0.835 ± 0.0165	**0.790** ± **0.0252**	**0.767** ± **0.0214**	**0.496** ± **0.0406**
XGBoost	0.577 ± 0.0984	0.685 ± 0.209	0.486 ± 0.325	0.567 ± 0.109	0.589 ± 0.0642	0.901 ± 0.439
LightGBM	0.618 ± 0.0480	0.207 ± 0.142	**0.962** ± **0.0347**	0.743 ± 0.263	0.308 ± 0.177	0.739 ± 0.0943
Tabnet	0.756 ± 0.0188	0.687 ± 0.117	0.814 ± 0.0763	0.764 ± 0.0454	0.715 ± 0.0502	0.521 ± 0.0223

Bold indicates the method with the best performance among all methods.

According to Table 4, for the causal network, using a smaller set of variables in the testing set cannot be better than using all variables. However, the performance using 12 variables in the Markov boundary equals to the performance using all variables in Table 3. In contrast, the performances of data-driven methods significantly decline. For the validation C using the testing sets with the complementary set of the Markov boundary, its result can be referred to in Table 5.

**Table 5:** Performance comparison for validation C

Method	Accuracy	Sensitivity	Specificity	Precision	F1-score	Log loss
Causal network	**0.619** ± **0.0145**	0.609 ± 0.0215	0.629 ± 0.0324	0.578 ± 0.0317	0.592 ± 0.0145	**0.635** ± **0.00827**
XGBoost	0.500 ± 0.0414	**0.921** ± **0.118**	0.149 ± 0.171	0.479 ± 0.0296	**0.625** ± **0.0195**	1.341 ± 0.267
LightGBM	0.546 ± 0.00220	0.00510 ± 0.00430	**0.998** ± **0.00220**	**0.633** ± **0.399**	0.0101 ± 0.00850	1.230 ± 0.0366
Tabnet	0.521 ± 0.0428	0.623 ± 0.298	0.435 ± 0.295	0.481 ± 0.0452	0.510 ± 0.140	0.711 ± 0.0402

Bold indicates the method with the best performance among all methods.

The result in Table 5 also shows that there are significant performance declines for data-driven models. And the proposed model in this work achieves the best performance comprehensively.

Conclusively, according to validation B and C, the proposed model shows its superiority to deal with the limited variable information. This indicates that the consideration of causality makes the proposed model has outstanding generalization and robustness under the data missing conditions. This is quite critical for the AMI diagnosis in the emergency department: even with part of the variables’ information, especially the Markov boundary for the onset of AMI, the causal network can support the effective and stable diagnosis.

### Causal effect quantification and verification

The causal effects between different variables and the onset of AMI are all quantified by EI. The EIs larger than 0 are listed as Table 6, where EI(*X*→AMI) represents the EI that results from other variables and leads to AMI, and EI(AMI→*X*) represents the EI that results from AMI and leads to other variables.

**Table 6:** EI related to AMI in the causal network

EI(*X*→AMI)		EI(AMI→*X*)	
Variable	EI	Variable	EI
DYS	0.0885	chest pain	0.0717
CD	0.0366	STSA	0.0291
CVD	0.0271	dyspnea	0.0274
Nutrition	0.0103	HGB	0.0246
Lifestyle	0.00255	numbness	0.0158
CRI (chronic renal insufficiency)	0.00146	TWA	0.0127
Sex	0.00100	ALB	0.00731
HTN	0.000238	–	–
Age	0.0000764	–	–

**Figure 6:**
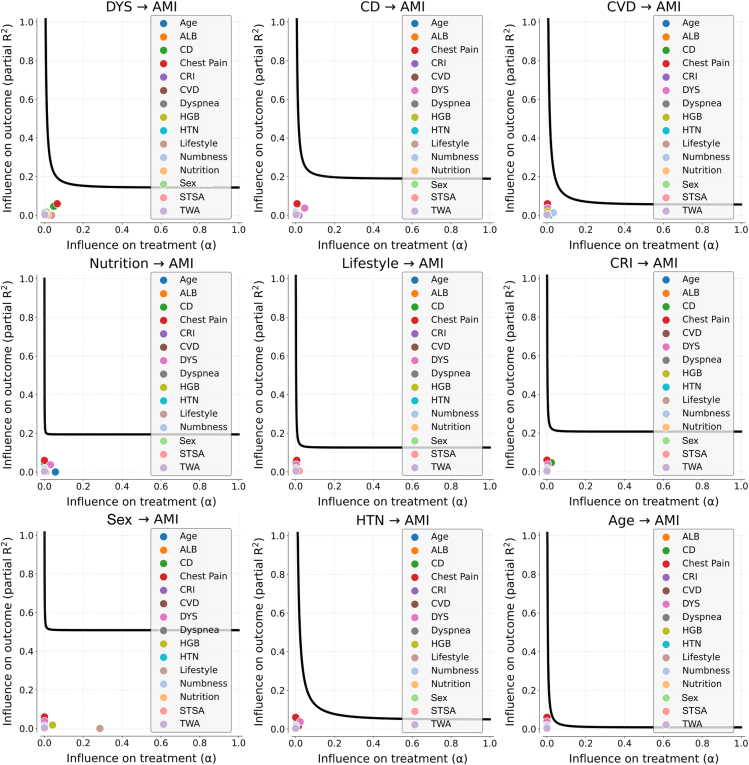
Sensitivity analysis for the variables with EI(*X*→AMI) > 0 Each subfigure is the Austen plot to test the potential unobserved confounding effect on one specific causal relationship marked in the subfigure’s title. The dots in an Austen plot present the effects of observed covariates. The curve indicates the effect of potential unobserved confounding bias. If the dots are closer to zero than the curve, it suggests that the causal relationship is robust. AMI, acute myocardial infarction. Full name of variables in subfigures can be referred to in Table 2.

**Figure 7:**
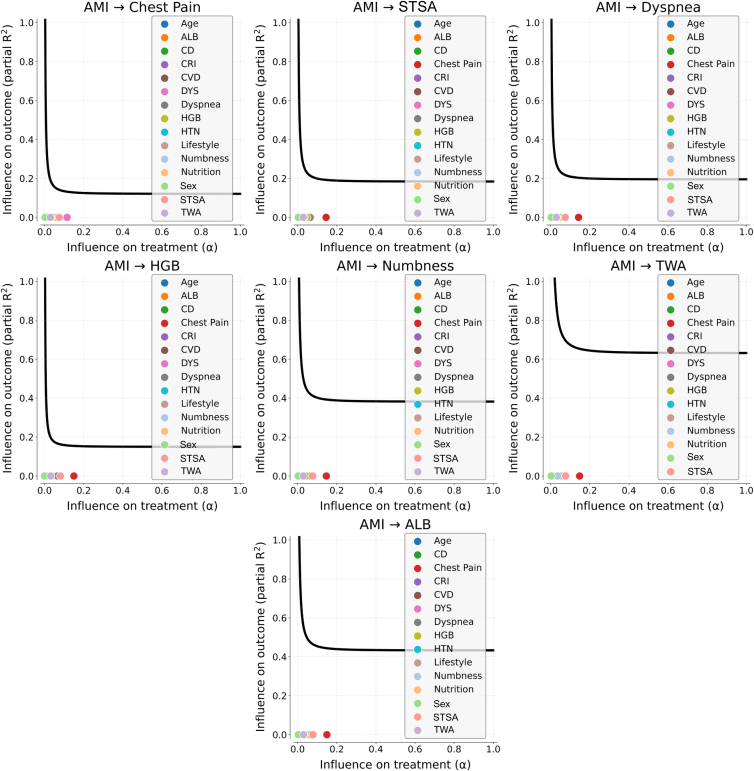
Sensitivity analysis for the variables with EI(AMI→*X*) > 0 Each subfigure is the Austen plot to test the potential unobserved confounding effect on one specific causal relationship marked in the subfigure’s title. The dots in an Austen plot present the effects of observed covariates. The curve indicates the effect of potential unobserved confounding bias. If the dots are closer to zero than the curve, it suggests that the causal relationship is robust. AMI, acute myocardial infarction. Full name of variables in subfigures can be referred to in Table 2.

For each variable in Table 6, a sensitivity analysis using Austen plot is performed to identify the potential unobserved confounding bias. The influences of confounders are studied when the potential bias in the average treatment effect (ATE) is supposed as a small value, i.e., 0.1, and they are compared with the influences of other covariates in Table 6. The Austen plot for each variable is depicted in Figures 6 and 7.

It can be figured that for all variables in Table 6, inducing a small bias requires the unobserved confounders with larger effects than other observed covariates, which suggests that the causalities are relatively robust. Also, the causal effects with AMI in Table 6 all were proved from the aspect of clinical knowledge (see details in Tables S2 and S3), which also supports the rationality of the discovered causalities.

## Discussion

### Comparison with standard clinical indicators

According to the 4th universal definition,^1^ AMI can be used to describe the condition when cTn significantly elevates or changes, and there is clinical evidence of myocardial ischemia such as typical chest pain or ECG features. For the included EMRs in this study, the statistics of cTnI, chest pain, and STSA are particularly listed in Table 7.

**Table 7:** Statistics of cTnI, chest pain, and STSA in the collected data

	cTnI = 1; Chest pain = 1 or STSA = 1^a^	cTnI = 1; Chest pain = 0 and STSA = 0	cTnI = 0; Chest pain = 1 or STSA = 1	cTnI = 0; Chest pain = 0 and STSA = 0
No AMI	15	208	508	2,539
AMI	16	27	1,658	1,030

a cTnI = 1 means that cTnI elevates; cTnl = 0 means no elevation. chest pain = 1 means that chest pain presents; chest pain = 0 means chest pain absents; STSA = 1 indicates that abnormal ST segment presents; STSA = 0 indicates that abnormal ST segment absents.

From Table 7, it can be concluded that 37.72% (1,030/2,731) of the patients with an ultimate diagnosis of AMI were not detected with an elevated cTnI, chest pain, or abnormal ST segment. Further, the AMI diagnosis performance of standard can be calculated, as shown in Table 8.

**Table 8:** AMI diagnosis performance of standard based on the collected data

Method	Accuracy	Sensitivity	Specificity	Precision	F1-score	Log loss
Standard	0.455	0.0049	**0.995**	0.516	0.0097	–
Causal network	**0.794** ± **0.0190**	**0.746** ± **0.0271**	0.835 ± 0.0165	**0.790** ± **0.0252**	**0.767** ± **0.0214**	**0.496** ± **0.0406**

Bold indicates the method with the best performance among all methods.

From Table 8, simply using cTnI, chest pain and ST segment achieves an unexpected performance. One main reason is that many patients with an ultimate diagnosis of AMI may not be detected with an elevated cTnI when they seek medical attentions. This phenomenon also has been stated by an observational study in a UK General Hospital’s emergency department^6^: 30.43% (28/92) of the patients with severe coronary artery stenosis were not detected with elevated cTn; by contrast, 40% (26/65) of the patients without severe coronary artery stenosis had elevated cTn. Therefore, only depending on cTn for AMI diagnosis may be limited in the emergency department. Potential reasons may be as follows:

(1) The dynamic change in cTn is regarded as one criterion for AMI diagnosis. After myocardial cell necrosis, the cTn level undergoes a process of “increase-maintenance-decrease.” In particular, cTn may not exhibit a rapid increase and reach a level detectable by cTn assays until several hours after myocardial cell necrosis.^31^ And this process is significantly affected by blood flow, leading to individual differences.^32^ Further, for the cTnI in the collected EMRs, its rise may be less significant due to the smaller cytosolic pool.^33^ Therefore, many patients with an ultimate diagnosis of AMI may not be detected with an elevated cTn when they seek medical attentions,^31^ considering the incomplete necrosis of myocardial cells, the delayed rise of cTn, or other individual factors. For this issue, the 4th universal definition also suggests that multiple follow-up detections should be performed after the initial cTn sampling, aiming to accurately diagnosis AMI. However, due to the practical constraints in the emergency department, it is common that only single cTn detection result is available for each patient, lacking the follow-up results. This may ignore the actual elevation of cTn, thus affect the causal characterization between cTn and AMI.
(2) cTn is organ-specific but not disease-specific.^31^ That is, cTn can be released as a result of ischemic, non-ischemic, and extra-cardiac conditions. Some study has recorded that ∼20% of patients may have elevated cTn, although most of them did not have ACS.^34^ Besides AMI, cTn elevations also can be observed in patients with chronic heart failure, kidney disease, diabetes, and even after extreme exercise.^31^ This may also affect the identification of causality between cTn and AMI.

Considering the potential atypical results of cTn, clinical guideline also strengthens that cTn measurements are not required for the initial stratification of ACS, and the initial emergency management should not be delayed even there is no elevation or changing of cTn.^20^ According to the findings in our study, we also hold that the diagnostic value of cTn should be treated with caution.

It should be strengthened that the proposed method in this study does not aim to deny or replace the standard method, but give more insights to deal with the challenge for the AMI diagnosis in the emergency department. Considering the atypical manifestations and limited information, discovering the causality between multiple variables is expected to make sense in AMI diagnosis.

### A comparative study based on MIMIC-IV-ED

To demonstrate the generalizability of the proposed causal method for AMI diagnosis in the emergency department, MIMIC-IV-ED^35^ is adopted, which contains the EMRs of the emergency department at the Beth Israel Deaconess Medical Center between 2011 and 2019. MIMIC-IV-ED contains six tables: edstays, diagnosis, medrecon, pyxis, triage, and vital sign. Table edstays records the patient demographics including race and sex, and the information such as admission time and discharging time; Table diagnosis records the diagnosis results with ICD codes; Table medrecon and pyxis record the information of medication use; Table triage and vital sign record the basic examinations such as respiratory rhythm (ResRhy) and systolic blood pressure (SBP), and the chief complaints containing symptoms.

**Figure 8:**
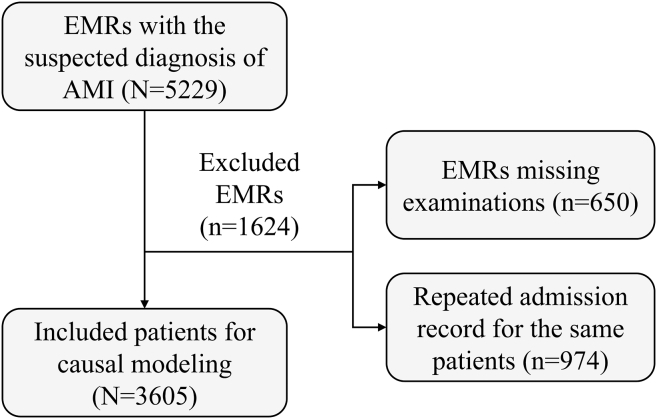
The flowchart to include the studied cohort of MIMIC-IV-ED EMR, electronic medical record; AMI, acute myocardial infarction.

A total of 5,229 EMRs are selected with the suspected diagnosis or symptoms. Also, the records with missing examinations and the repeated admission records for the same patients are excluded. The exclusion flowchart can be referred to in Figure 8. Finally, 3,605 EMRs are included, in which 1,462 patients were diagnosed with AMI.

For the considered variables, MIMIC-IV-ED did not record past medical history, lifestyle, biochemical profile, and ECG. Therefore, referring to Table 1, five variables are included for each EMR, which can be divided as follows: (1) sex recorded in Table edstays; (2) ResRhy and SBP recorded in Table triage; (3) chest pain, numbness, and dyspnea recorded in the chief complaints of Table triage. In addition, whether the patient is diagnosed with AMI is recorded in Table diagnosis and included in this study. Statics of the included variables can be referred to in Table 9.

**Table 9:** Statistics of the included variables in the comparative study

Category	Variable	Discretization criteria	AMI = 0 (2,143)	AMI = 1 (1,462)	***χ***^2^	*p* value
Demography	sex	0: female	983	621	4.054	0.0441
		1: male	1,160	841		
Symptom	chest pain	0: no	1,586	776	168.531	<0.0001
		1: yes	557	686		
	limb numbness (numbness)	0: no	2,141	1,460	0.148	0.7003
		1: yes	2	2		
	dyspnea	0: no	1,803	1,412	139.529	<0.0001
		1: yes	340	50		
Other examine	systolic blood pressure (SBP) (mmHg)	0: <90	27	26	7.246	0.02670
		1: 90-140	1,154	841		
		2: >140	962	595		
	respiratory rhythm (ResRhy) (breaths/min)	0: <10	2	2	30.165	<0.0001
		1: 10-24	1,994	1,420		
		2: >24	147	40		

**Figure 9:**
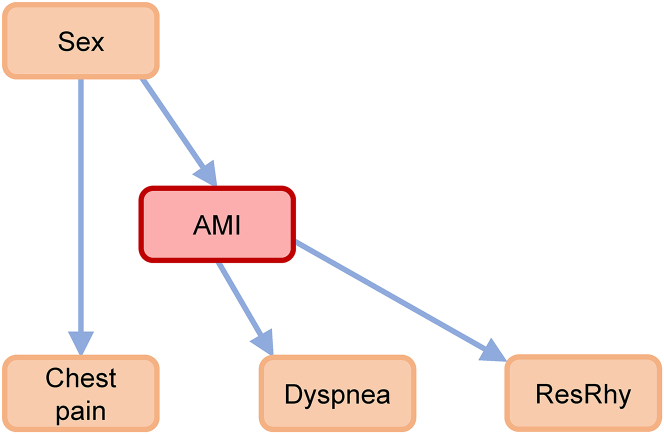
Causal network based on the cohort in MIMIC-IV-ED The causal network contains 5 variables, with all edges determined. The causalities related to numbness and systolic blood pressure (SBP) cannot be identified. ResRhy, respiratory rhythm.

FGES is adopted to construct MEC under constraints. The result is illustrated in Figure 9. With the established causal network, validation A is performed. Table 10 shows the performances of causal network and data-driven methods.

**Table 10:** Performance comparison based on the cohort in MIMIC-IV-ED

Method	Accuracy	Sensitivity	Specificity	Precision	F1-score	Log loss
Causal network	0.642 ± 0.0247	0.452 ± 0.0407	0.771 ± 0.0359	0.575 ± 0.0507	**0.505** ± **0.0388**	0.634 ± 0.0183
XGBoost	0.641 ± 0.0284	**0.453** ± **0.0606**	0.770 ± 0.0335	0.573 ± 0.0451	0.504 ± 0.0507	0.634 ± 0.0176
LightGBM	0.642 ± 0.0285	0.452 ± 0.0598	0.771 ± 0.0317	0.574 ± 0.0449	0.505 ± 0.0507	0.634 ± 0.0178
Tabnet	**0.644** ± **0.0120**	0.432 ± 0.0720	**0.789** ± **0.0592**	**0.593** ± **0.0601**	0.492 ± 0.0498	**0.632** ± **0.00890**

Bold indicates the method with the best performance among all methods.

Given all variable information for testing sets, the performance for AMI diagnosis of causal network is still similar with data-driven methods. However, the diagnostic performance using only four variables in MIMIC-IV-ED is significantly lower than the performance using multiple variables from the dataset collected by authors.

For the included variables in this case, the demographics of sex and the symptoms of chest pain and dyspnea can be easily clarified in clinic, while ResRhy needs to be specifically detected. Therefore, the validation using part of variables is also performed for this case, in which training set contains all variables while testing set misses the information of ResRhy. Performance results can be referred to in Table 11.

**Table 11:** Performance comparison based on the cohort in MIMIC-IV-ED, with part of variables

Method	Accuracy	Sensitivity	Specificity	Precision	F1-score	Log loss
Causal network	**0.640** ± **0.0247**	**0.458** ± **0.0396**	0.764 ± 0.0372	0.570 ± 0.0517	**0.507** ± **0.0382**	**0.635** ± **0.0170**
XGBoost	0.595 ± 0.00230	0.0212 ± 0.0671	**0.987** ± **0.0427**	0.0517 ± 0.163	0.0301 ± 0.0952	0.741 ± 0.0208
LightGBM	0.640 ± 0.0289	0.458 ± 0.0551	0.763 ± 0.0363	**0.570** ± **0.0462**	0.507 ± 0.0467	0.636 ± 0.0187
Tabnet	0.634 ± 0.0262	0.395 ± 0.128	0.797 ± 0.0568	0.567 ± 0.0351	0.456 ± 0.107	0.639 ± 0.0158

Bold indicates the method with the best performance among all methods.

According to Table 11, it suggests that under the data missing condition, the proposed causal network achieves the best performance comprehensively, similar with the results from the dataset collected by authors.

Besides the diagnosis performance, the causal effects between AMI and other variables are quantified by EI, and the results are listed in Table 12.

**Table 12:** EI related to AMI in the causal network of MIMIC-IV-ED

EI(*X*→AMI)		EI(AMI→*X*)	
Variable	EI	Variable	EI
Sex	0.000568	dyspnea	0.0237
		chest pain	0.0230
		resRhy	0.00471

According to Table 12, the relatively large causal effects related to chest pain and dyspnea can be identified, similar to the results in Table 6. Also, a less effect on ResRhy can be observed. However, the effect on numbness is not significant. This may be because of the following reasons:

(1) For this case, the symptoms like numbness are exacted from the chief complaints in Table triage. The chief complaints are extremely brief, consisting of a few terms. By contrast, the symptoms in the dataset collected by authors are identified from the detailed descriptive texts in EMRs. This may lead to the difference between the results of two datasets.
(2) There could be inevitable selection bias due to the differences in the patients’ races. The dataset collected by authors describes the patients in North China, while MIMIC-IV-ED includes the patients in the USA. This may also affect the causality identification.

Conclusively, the proposed causal network also can be applied in the cohort of MIMIC-IV-ED. Causal network achieves the better diagnostic performance given limited variables, compared with prominent data-driven methods. It also suggests that a cohort with multiple variables could show better performances in AMI diagnosis. In addition, the causal effects related to AMI can be quantified. It shows that AMI has relatively large effects with chest pain and dyspnea, similar to the results of the data collected by authors.

### Support for clinical decision making

The established causal network is expected to support clinical decision-making. When used in clinic, the computational latency should be focused. The experiments in this work are performed on an Intel i7-10750H CPU with 12 cores. A 10-fold random cross-validation including training and testing requires around 5 min, and one AMI diagnosis based on the trained model only requires less than 0.01 s, which can meet the efficiency demand in the emergency department. In addition, the Markov boundary of AMI is identified, reducing the number of necessary variables for maintaining optimal diagnostic performance to 12. This suggests a simplified priority checklist to optimal the triage protocols in the emergency department: in clinic, it could be recommended to preferentially identify these variables, which will further shorten the time to obtain variable information and effectively diagnosis AMI. Meanwhile, it also should be acknowledged this Markov boundary is still a suggestion. Large-scale, multi-center studies and necessary experimental verifications are required before any guidelines can be formulated.

In addition, the established model shows its superiority in interpretability, especially compared with data-driven methods. Clinicians can visually understand the causal paths of AMI onset. For instance, the typical path that DYS may lead to AMI can be identified, which support the AMI diagnosis and prevention in clinic. On this basis, the causal effects between AMI and multiple variables are measured. Causal effects reflect the potential results after interventions, which also support disease treatment and healthcare.

(1) The reasons leading to AMI (the variables with EI(*X*→AMI)) suggest that the past history of DYS and cardio-CVDs are critical risk factors of AMI. Therefore, effective lipid management and cardio-cerebrovascular healthcare in daily life may help to prevent AMI.
(2) The results caused by AMI (the variables with EI(AMI→*X*)) include clinical symptoms (i.e., chest pain, dyspnea, and numbness), and routine examinations like blood routine (i.e., HGB and ALB) and ECG (i.e., STSA and TWA). This indicates the importance of such symptoms and examinations for the AMI diagnosis in the emergency department.

Further, since the causal network modeling is with clinical practical significance, this also help clinicians build trust in the model. Variables are discretized based on clinical guidelines, and prior knowledge constraints are introduced to ensure the rationality of modeling. Also, the causal effects with AMI all have been proved by existing medical studies, which also support the interpretability of the proposed method.

### Limitations of the study

There are still some limitations that should be strengthened. In this work, causal network is adopted for AMI diagnosis in the emergency department, rather than ACS, or the subtypes of AMI, i.e., ST-segment elevation myocardial infarction (STEMI) and non-ST-segment elevation myocardial infarction (NSTEMI). The reason that we focus on AMI instead of ACS is to accurately identify the patients with severe conditions in the emergency department. ACS includes AMI and unstable angina (UA), and these two subtypes are significantly different in clinical manifestations and treatment strategies. Thus, a general diagnosis of ACS is not appropriate. UA is a relatively mild disorder caused by transient myocardial ischemia, and myocardial cell necrosis generally does not occur in this case. The treatment of UA aims to relieve myocardial ischemia and prevent the progression to AMI, with a relatively favorable prognosis. In contrast, AMI is characterized by the presence of myocardial cell necrosis, with a far higher mortality risk than UA. PCI and other interventional measures are usually required for reperfusion, and the time window for urgent treatment is much narrower. Therefore, in the emergency department, it is necessary to further identify AMI patients from the ACS population, so as to promptly assess risk and initiate treatment.

In addition, the reasons that we choose AMI without further distinguishing STEMI and NSTEMI mainly include the following:

(1) This classification that uses clinical manifestations as a proxy for pathology may lead to misdiagnosis of patients’ actual condition. The American college of cardiology expert consensus states that “the application of STEMI ECG criteria on a standard 12-lead ECG alone will miss a significant minority of patients with acute coronary occlusion (ACO).”^36^^,^^37^ In particular, in a meta-analysis conducted by Avdikos et al., 25.5% of patients diagnosed as NSTEMI were found to have ACO, which results in a delay in reperfusion therapy.^38^ Further, randomized studies have found lower mortality in NSTEMI patients who received immediate reperfusion.^39^ It suggests that the therapeutic needs of NSTEMI and STEMI are comparable, and there may not be strict boundary between NSTEMI and STEMI.
(2) Since coronary occlusion is a progressive process, ST segment also exhibits dynamic change. This also indicates that a single ECG assessment may not accurately reflect the patient’s coronary artery status. As a result, clinical guidelines also recommend clarifying STEMI and NSTEMI based on the persistent ST segment elevation in continuous ECG monitoring.^20^ However, due to the narrow time window, it is sometimes difficult to conduct sufficient continuous ECG monitoring in the emergency department.

Considering the controversial criterion and practical challenge to distinguish STEMI and NSTEMI, it is common that clinicians merely rendered a diagnosis of AMI without conducting any further subclassifications in the emergency department. Combining the above reasons, in this work, we focus on AMI rather than ACS, STEMI, or NSTEMI.

Besides the studied disease, it should be noted that the inclusion and utilization of real EMRs affect the modeling and conclusions. For the dataset collected by authors, only the patients in the years of 2021 and 2022 from Beijing Jishuitan Hospital, Beijing, China are studied. Although the collected data are from three different campuses located in two districts of Beijing, it still may lead to inevitable selection bias. Conservatively, the model and conclusions may be best applicable to the population in North China. In addition, the problems in data quality such as uncontrollable measurement errors and entry mistakes also affect the accuracy of AMI diagnosis and conclusions. However, as mentioned in introduction, causal network is more robust against data noise from the modeling principle. In addition, prior medical knowledge constraints are integrated in the causal modeling. This can filter out the false associations caused by data noise, thereby reducing the impact of data noise on the causal structure. According to the model validation with data missing condition and the sensitivity analysis on unobserved confounding bias, causal network can maintain a stable diagnostic performance against data noise. Considering the life-critical nature of hospitals, there should be strict quality control in EMRs. Consequently, the data noise in real EMRs could be mild, as well as its effect on the diagnostic performance.

For each patient, according to clinical knowledge and expert experience, 54 variables are included, while the appropriateness of including these variables is also worth discussing: the included variables may increase the burden of data collection and management in clinical practice. For this point, in fact, the Markov boundary and the variables with causal effects related to AMI have been identified. This dimensionality reduction supports to concentrate on necessary variables when making decisions for AMI diagnosis, treatment, and healthcare.

From another side, there may also be some variables contributing to the AMI diagnosis in the emergency department that have not been considered. For instance, the dynamic time series variables are not included, considering the data availability of EMRs. Although we mainly focus on the time window when patients are admitted in the emergency department, it should be noted that the onset of AMI in the emergency department also has a temporal progression, especially for atypical patients. It is challenging but valuable to consider the temporal dynamics for data management and causal modeling in the future work.

In addition, in this work, the network coarse-graining is proposed to obtain a unique and qualified causal network, which may lead to the inaccessibility of some information under the macro variable. For this case, it is suggested to search the minimum set of variables for coarse-graining; also, the medical knowledge should be concerned to make the macro variable explainable.

For the discovered causality related to the onset of AMI in this work, it should be emphasized that since it is inferred based on SCM and observed data, the causality is essentially a suggestion for potential mechanisms and knowledge. Although the sensitivity analysis on unobserved confounding bias is performed and corresponding medical knowledge has been reviewed, for the discovered causality from data, in-depth and rigorous clinical trial validations are still the future work need to be performed.

Conclusively, to solve the clinical challenge of AMI diagnosis in the emergency department, in this work, the causality related to the onset of AMI is studied based on real EMR data, and the causal network can be constructed for AMI diagnosis. The following conclusions can be drawn.

(1) An effective framework to construct the causal network for AMI diagnosis based on real EMR data is proposed, in which the causalities among multicategory variables can be modeled by the score-based algorithm, SEM, and network coarse-graining.
(2) A model validation procedure is proposed to test the model performance when only part of variables’ information can be obtained. According to the validation, the established causal network shows its superiority compared with the prominent data-driven methods. Not limited to AMI, the model validation procedure can be generalized for other disease diagnostic models.
(3) The causal effects between variables and the onset of AMI can be quantified, and the sensitivity analysis is performed to verify the robustness of the causality against the unobserved confounding bias. The causality figures out the critical risk factors of AMI, and indicates the importance of clinical symptoms and routine examinations for the AMI diagnosis in the emergency department.

For the future work, the proposed method is expected to integrate with clinical workflows. The key variables identified in this work (such as the Markov boundary of AMI) can improve data collection procedure. Also, the model can be deployed in the information systems to provide assistance in clinic. In addition, the discovered causalities from observed data should be verified according to clinical trials, and the causalities covered by coarse-graining should be further studied. Also, the daily health data can be collected by wearable devices (e.g., monitoring heart rate and physical activity via smart watches). Integrating multi-center electronic health records and follow-up data, the dataset containing daily health status, disease development, and rehabilitation process can be established. The subjects’ variables from different time phases can be incorporated to construct a causal network with the spatiotemporal information during the whole disease period. Further, not limited by disease diagnosis, the proposed method is expected to be applicable for the guidance to identify potential interventions in the public health field, enhancing the value in interdisciplinary fields.

## Resource availability

### Lead contact

Requests for further information and resources should be directed to and will be fulfilled by the lead contact, Xiao-Yang Li (leexy@buaa.edu.cn).

### Materials availability

This study did not generate any new materials.

### Data and code availability

• The structural data processed from real EMRs, the established causal network, and the code for AMI diagnosis, model validation, causal effect quantification, and verification are all available at Github: https://github.com/modestmason/Causal-network-analysis-for-AMI-diagnosis-in-the-emergency-department.
• The tool to build MEC is available at: https://www.cmu.edu/dietrich/philosophy/tetrad/.
• The code for DANM is available at: https://webdav.tuebingen.mpg.de/causality/.
• Any additional information required to reanalyze the data reported in this paper is available from the lead contact upon request.

## Acknowledgments

This work is supported by the Beijing Natural Science Foundation of China (grant no. 7262060) and (grant no. 7222086), and the 10.13039/501100001809National Natural Science Foundation of China (grant no. 51775020).

## Author contributions

Conceptualization, B.-Y.L., X.-Q.L., Y.-T.J., X.-Y.L., Z.-X.T., and R.K.; methodology, B.-Y.L., X.-Q.L., Y.-T.J., X.-Y.L., and Z.-X.T.; investigation, B.-Y.L., X.-Q.L., and Y.-T.J.; writing – original draft, B.-Y.L.; writing – review and editing, B.-Y.L., X.-Q.L., Y.-T.J., X.-Y.L., and Z.-X.T.; funding acquisition, X.-Y.L. and R.K.; resources, X.-Q.L., X.-Y.L., and Z.-X.T.; supervision, X.-Y.L. and R.K.

## Declaration of interests

The authors declare no conflict of interest.

## STAR★Methods

### Key resources table

**Table:** 

REAGENT or RESOURCE	SOURCE	IDENTIFIER
**Deposited data**		

The structural data processed from real EMRs, the established causal network, and the code for AMI diagnosis, model validation, causal effect quantification and verification	Github	Github: https://github.com/modestmason/Causal-network-analysis-for-AMI-diagnosis-in-the-emergency-department

**Software and algorithms**		

Python version 3.8	Python Software	https://www.python.org/
Sklearn version 1.0.2	Scikit-learn Software	https://scikit-learn.org/stable/api/sklearn.html
Pandas version 1.4.2	Pandas Software	https://pandas.pydata.org/
Numpy version 1.21.5	NumPy Software	https://numpy.org/
math	Python Software	https://docs.python.org/3/library/math.html
Causalnex version 0.11.0	Github	Github: https://github.com/mckinsey/causalnex
Tetrad version 7.5.0	Tetrad Software	https://www.cmu.edu/dietrich/philosophy/tetrad/
DANM	Dr. Jonas Peters	https://webdav.tuebingen.mpg.de/causality/

### Experimental model and study participant details

The real EMRs in the emergency department with multicategories and large samples are adopted in this work. In particular, the anonymized EMRs in the emergency department of Beijing Jishuitan Hospital, Beijing, China are collected by the authors’ team. Based on ICD-10 diagnosis codes, 7918 EMRs in the years of 2021 and 2022 are collected, which were all with the suspected diagnosis of ischemic heart diseases (I20.004, I20.102, I20.804, I20.902, I21.201, I21.304, I21.403, I21.902, I22.901, I24.803, I24.901, I25.104, I25.105, I25.210, I25.501, I25.601) or potential symptoms of AMI (R06.001, R07.401, R09.896, R11, R42, K27.903).

Further, the EMRs are excluded based on the following criteria: (1) exclude the records without biochemical profiles; (2) for the patients who repeat admitted to the emergency department during this period, since the repeated samples from one individual may affect the diagnostic model training, only the EMRs of their first admissions are considered. The flowchart to include the studied cohort is presented in Figure 1. Conclusively, 6001 patients are included in this work, in which 2731 patients were finally diagnosed with AMI. This study was approved by the Bioethics and Medical Ethics Committee of Beihang University (No. BM20240031). As a retrospective study, no animal experiment or clinical trial is performed. The structural data processed from real EMRs including sex and age is available, while it should be acknowledged that gender, ancestry, race, and ethnicity were not recorded in the collected EMRs, of which the influences may affect the conclusions. Details can be referred to section resource availability.

### Method details

#### Medical knowledge constraints setting

The causal modeling among diverse variables considers the prior constraints based on medical knowledge to ensure the rationality of modeling. Specifically, two aspects of medical knowledge constraints are considered.

(1) Forbid the causalities within demography, and those within clinical manifestations including symptom, biochemical profile, ECG, and other examine.

In EMRs, the causalities within demography such as age and sex should be forbidden. The wrong causalities within demography often result from the selection bias in data collection, which should be avoided. In addition, symptoms, biochemical profiles, ECG, and other examines are the external manifestations in relatively short time, which are all caused by some underlying reasons such as the onset of AMI. In this work, the causalities within clinical manifestations are not considered.

(2) Forbid the causalities against the temporal order.

From the perspective of temporal order, the variables that occur later cannot be the reasons of those occurring earlier. For instance, the categories such as symptom and biochemical profile should be the results of AMI. And some categories including lifestyle and past medical history occur before the onset of AMI. Specifically, the concerned categories in the EMRs of AMI patients follow the temporal order as: demography → lifestyle → past medical history → the onset of AMI → clinical manifestations including symptom, biochemical profile, ECG, and other examine. The causalities against the temporal order should be forbidden.

#### Score-based Markov equivalence class construction

In SCM, the methods to identify causalities can be mainly divided into the constraint-based methods and the score-based methods. In addition, some methods discover causalities based on time series. However, for the causal discovery related to the onset of AMI in the emergency department, the constraint-based methods are generally time-consuming to deal with a large number of variables in EMRs, and there is no time series to support the time series-based methods in this work. By contrast, the score-based causal discovery algorithms can effectively construct the MEC from a large number of variables in EMRs, without relying on time series. In this work, one typical and prominent method of the score-based algorithms, FGES,^21^ is adopted, and the medical knowledge constraints are considered.

FGES is a parallelized score-based algorithm, which can construct the MEC effectively by optimizing the Bayesian information criterion (BIC) as $\text{BIC}=2L-k\;\ln\;N,L=\sum_{i=1}^{n}\ln\left(\mathrm{Pr}\left\{X_{i}|pa\left(X_{i}\right)\right\}\right)$, where: L is the likelihood function; *X*_*i*_ is the *i*^th^ variable, and *n* is the total number of variables; *pa*(*X*_*i*_) is the parents of *X*_*i*_, and *Pr*{*X*_*i*_|*pa*(*X*_*i*_)} is estimated by observation data and the candidate graph; *k* is the number of parameters that increases monotonically with the number of edges, and *N* is the sample size.

FGES starts with an empty graph, and adds the edges which maximizes the BIC score; then the edges are tested and possibly eliminated to increase the BIC score. The BIC score is iteratively optimized and the final MEC can be obtained. The basic logic of FGES is that.

(1) Begin with an empty graph;
(2) Rank to obtain the directed edge list *L* ordered by the BIC score of graph when adding each edge;
(3) Add the edge with the rules that: (a) the BIC score of the graph is optimally improved; (b) the graph after adding edges should follow medical knowledge constraints and no new cycle is introduced. If a new V-structure generates within three nodes after adding the edge, reorient undirected edges between each pair of the three nodes to avoid false dependence. Rescore the potential edges based on the updated graph, and reform the edge list *L*;
(4) Repeat step 3 until *L* is empty or no edge can satisfy the rules in step 3;
(5) Search and delete the edges that can optimize the score, and orient edges using Meek’s rules, ensuring that the graphs before and after update belong to the same MEC;
(6) Repeat step 5 until the score cannot be optimized.

In particular, the Meek’s rules^40^ mentioned in step 5 aim to deduce network topology from independence.

(1) For *A*→*B* and *B*—*C* with *A* and *C* being non-adjacent, orient *B*→*C*;
(2) For *A*→*B*, *B*→*C*, and *A*—*C*, orient *A*→*C*;
(3) For *A*→*B*, *A*→*C*, *B*—*C*, *B*→*D*, and *C*—*D*, orient *C*→*D*.

#### Causal network identification based on structural equation model and network coarse-graining

For the constructed MEC, an edge is built for a better BIC score, and if it violates original conditional independence or cannot be judged by orientation rules, it will be undirected. While the correlations between variables can be inferred from the BIC score optimization, the causal direction cannot be solely determined by conditional independence.

When we try to clarify the directions of these edges, one necessary premise is that the network after setting directions also should belong to the former constructed MEC, corresponding to the conditional independence in data. Therefore, the newly determined directions should not introduce new V-structures or cycles.

##### SEM-based causal direction identification

Firstly, SEM can be adopted to construct the functions between two variables and capture the asymmetric generative relationships, if no new V-structure or cycle generates. Specifically, SEM fits the function containing variables and the noise like *Y* = *f*(*X*,*ε*; θ), where: *X*, *Y* are variables; *f*(·) is the function explaining how *Y* is generated from *X*; *ε* is the noise independent of *X*; and θ is the parameter vector.

To deal with the discrete variables in EMRs, the discrete nonlinear additive noise model (DANM) is specifically adopted.^41^ DANM proposed the discrete regression method to construct the nonlinear function between discrete variables. Then the correlations between variables and residual errors can be measured, and the causal directions can be identified. The discrete regression method of DANM can be concluded that.

(1) Initialize $f^{\left(0\right)}\left(x_{i}\right)={\mathrm{argmax}}_{y}\hat{P}\left(X=x_{i},Y=y\right)$, *j* = 0, where *x*_*i*_ is the *i*^th^ value of *X*.
(2) *j* = *j* + 1. Then for each *i*, $f^{\left(j\right)}\left(x_{i}\right)={\mathrm{argmin}}_{y}\text{DM}\left(X,Y-f_{x_{i}\mapsto y}^{\left(j-1\right)}\left(X\right)\right)$, where $f_{x_{i}\mapsto y}^{\left(j-1\right)}\left(X\right)$ means *f*^(*j*−1)^ but *f*(*x*_*i*_) = *y*, and *y* equals to ${\mathrm{argmax}}_{y}\hat{P}\left(X=x_{i},Y=y\right)$. DM is the negative *p*-value of the Pearson’s Chi-square test, which measures dependence.
(3) Repeat step 2 until the residual error $\hat{\varepsilon }=Y-f^{\left(j\right)}\left(X\right)$ is independent with *X* (tested by the Pearson’s Chi-square test), or *f*^(*j*)^ does not change.

##### Network coarse-graining

If the directions still cannot be identified by SEM, or new V-structures or cycles must generate when setting directions, it may suggest that there are bidirectional causalities or unobserved confounders, which are challenging to study. For this case, the related variables and edges can be integrated as a new macro variable, which is essentially the network coarse-graining to avoid newly generated V-structures or cycles. The specific steps for the coarse-graining can be summarized as follows, also as depicted in Figure 2C.

(1) Locate the uncertain edges, if the direction cannot be identified by SEM or new V-structures or cycles generate;
(2) For one unclear edge *E*, find a set of variables, which contains the variables at the ends of *E* and other necessary variables to avoid newly generated V-structures or cycles;
(3) Search the minimum set (Ω_*E*_) among all candidates, and integrate Ω_*E*_ as a macro variable *V*_*E*_. The value space of *V*_*E*_ is the combinations of the values of all variables in Ω_*E*_.
(4) Since *V*_*E*_’s states combine all variables’ states, the former causalities between the variables in Ω_*E*_ and those not in Ω_*E*_ also exist between *V*_*E*_ and the same variables not in Ω_*E*_.

According to the coarse-graining, the edges with uncertain directions can be covered under the macro variables, and the unique and qualified causal network can be obtained to support the following AMI diagnosis and analysis.

#### Model validation procedure

Based on the established causal network, the Bayesian estimation method is adopted for parameter learning, and the probability distribution for the onset of AMI can be inferred. For the AMI diagnosis, the probability classification threshold is set as 0.5.

To verify the diagnostic performance with data missing condition, three validations are performed, in which the training sets include all variable information, but the testing sets are different: validation A: the testing sets include all variables except the onset of AMI; validation B: the testing sets only include the Markov boundary of AMI; validation C: the testing sets only include the complementary set of the Markov boundary, also excluding AMI. The variables in each testing set can be referred as Figure 5.

The variables with missing values in the testing sets of validation B and C are regarded as hidden variables. When inferring the probability of AMI, the conditional probability distribution of AMI and hidden variables given other observed variables is first calculated using the estimated network parameters. On this basis, by summing over the probabilities of all possible states of hidden variables, the marginal distribution of AMI is solved to support AMI diagnosis.

For each validation, 6001 patients’ data are all included, thus the patients in three validations 100% overlap. For validation A, the patients’ data in the testing set contains all variables, same as those in the training set. Therefore, it is a benchmark to test the basic effectiveness for AMI diagnosis. For validation B and C, the data in the testing sets only contain part of variables. It corresponds to the condition that some variable information cannot be obtained when using the algorithm for AMI diagnosis in clinic. In particular, the causal network’s performance in the validation B is expected to be similar with that in the validation A.

#### Causal effect quantification and verification

To quantify causal effects, EI was proposed by Hoel et al.,^42^ which studies the difference caused by specific interventions and identifies the causalities. In particular, EI is the Kullback-Leibler divergence between the results under different interventions and the average result under the equal-probability intervention.

In this work, EI is adopted to quantify the causal effects in the causal network.^43^ For a pair of variables in the causal network, for instance, the onset of AMI (*A*) and chest pain (*C*), the EI from *A* to *C*, EI(*A*→*C*), can be calculated as follows:

$$\begin{array}{c} \text{EI}\left(A\to C\right)=\sum_{i=1}^{N_{A}}\mathrm{Pr}\left\{do\left(A=a_{i}\right)\right\}\times \sum_{k=1}^{N_{C}}\left(\mathrm{Pr}\left\{C=c_{k}|do\left(A=a_{i}\right)\right\}\times \ln\frac{\mathrm{Pr}\left\{C=c_{k}|do\left(A=a_{i}\right)\right\}}{\mathrm{Pr}\left\{C=c_{k}|U_{A}\right\}}\right)\\ \mathrm{Pr}\left\{C=c_{k}|U_{A}\right\}=\sum_{i=1}^{N_{A}}\mathrm{Pr}\left\{do\left(A=a_{i}\right)\right\}\times \mathrm{Pr}\left\{C=c_{k}|do\left(A=a_{i}\right)\right\},\mathrm{Pr}\left\{do\left(A=a_{i}\right)\right\}=\frac{1}{N_{A}} \end{array}$$

where: *do* represents the do-operator in SCM, which is calculated by do-calculus; *a*_*i*_ is the *i*^th^ state of *A*, and *N*_*A*_ is the total number of states of *A*; *c*_*k*_ is the *k*^th^ state of *C*, and *N*_*C*_ is the total number of states of *C*; and *U*_*A*_ represents the equal probabilistic intervention on *A*. The larger EI(*A*→*C*) is, the more significant the causal effect from the onset of AMI to chest pain is.

In clinic, the variables that have significant causal effects with the onset of AMI can provide valuable information for disease diagnosis, which should be preferentially examined. In addition, the control and improvement of some variables that significantly lead to the onset of AMI may be effective for the treatment and health management.

For the variables having causal effects with the onset of AMI, the sensitivity analysis is performed for each variable to clarify the potential unobserved confounding bias. For this point, a widely applied approach proposed by Veitch and Zaveri^44^ is adopted. For each causality to be verified, the influence of unobserved confounders on the cause is first evaluated, which introduces the assumption of Beta distribution and proposes the sensitivity parameter *α* to quantify the influence:

$$\tilde{g}\left(X,U\right)|X∼\text{Beta}\left(g\left(X\right)\left(1/\alpha -1\right),\left(1-g\left(X\right)\right)\left(1/\alpha -1\right)\right)$$

where: *X* is the covariate, and *U* is the unobserved confounder; *g*(*X*) is the propensity score, for a binary cause *T* valued as 0 or 1, *g*(*X*) = *Pr*(*T* = 1|*X*); $\tilde{g}\left(X,U\right)$ is the distribution of *g*(*X*) considering *U*; *α* is the sensitivity parameter valued between 0 and 1, and a larger *α* indicates a larger influence of the confounder on the cause.

For the influence of unobserved confounders on the outcome, based on logistic assumption, the expected outcome considering unobserved confounders can be provided as:

$$E\left[Y|T,X,U\right]=Q\left(T,X\right)+\delta \left(\text{logit}\;\tilde{g}\left(X,U\right)-E\left[\text{logit}\;\tilde{g}\left(X,U\right)|T,X\right]\right)$$

where: *Y* is the outcome; *Q*(*T*,*X*) is the conditional expected outcome without the consideration of unobserved confounders, and *Q*(*T*,*X*) = E[*Y*|*T*,*X*,]; *δ* is the sensitivity parameter reflecting the influence of unobserved confounders on the outcome; and logit means the logarithm operation.

The unobserved confounding bias when estimating the average treatment effect (ATE) can be derived:

bias=δE[ψ(g(X)(1/α−1)+1)−ψ((1−g(X))(1/α−1))−ψ(g(X)(1/α−1))+ψ((1−g(X))(1/α−1)+1)]

where *ψ*(·) is the digamma function.

Since *δ* is dimensional, the partial coefficient, $R_{Y,\text{par}}^{2}$, is adopted to replace *δ* to represent the influence on the outcome, which is regularized and unitless:

$$R_{Y,\text{par}}^{2}=\frac{E{\left(Y-Q\left(T,X\right)\right)}^{2}-E{\left(Y-E\left[Y|T,X,U\right]\right)}^{2}}{E{\left(Y-Q\left(T,X\right)\right)}^{2}}$$

*g*(*X*) and *Q*(*T*,*X*) can be estimated by statistical data, which can be substituted into the above equations to calculate the influences and bias. To clarify whether the potential bias is significant, the unobserved confounding bias should be compared with the influences of observed covariates. In particular, for one observed covariate *Z*, estimate *g*(*X*) and *Q*(*T*,*X*) assuming that *Z* is not considered, which can be calculated by the inference results of *T* and *Y* lacking the information of *Z*. Combined with *g*(*X*) and *Q*(*T*,*X*) considering *Z*, the *α* and $R_{Y,\text{par}}^{2}$ of *Z* can be estimated.

Compared with other methods, this approach does not depend on any assumption for the generation function from the cause to the outcome. On this basis, an Austen plot can be provided to illustrate the comparison between the influences of unobserved confounders and observed covariates. As shown in the Figure S1 in Supplemental Information, the x axis and y axis of the Austen plot represent *α* and $R_{Y,\text{par}}^{2}$ respectively. And the curve indicates the *α* and $R_{Y,\text{par}}^{2}$ of unobserved confounders when the unobserved confounding bias equals to some small value. Each dot in the plot represents the *α* and $R_{Y,\text{par}}^{2}$ of one observed covariate. If the dots are close to the origin compared with the curve, it suggests that the unobserved confounders need to have quite large influences on the cause and outcome to result in a small confounding bias, compared with observed covariates. Then we can assume that there are no unobserved confounders, and the causality is robust.

### Quantification and statistical analysis

Chi-square test is adopted to for comparisons between AMI patients and non-AMI patients, as the basic descriptive statistics for each variable.

For the validation A, B, and C to verify diagnostic performance, the 10-fold random cross-validation is adopted for data splitting in each validation. The entire dataset is randomly divided into 10 subsets. During each iteration of cross-validation, 9 subsets are combined as the training set (containing all variable information), and 1 subset serves as the testing set (containing part of variable information for validation B and C). This process is repeated 10 times, with each subset used once as the testing set.

The diagnostic performance is evaluated by typical metrics including accuracy, sensitivity, specificity, precision, F1-score, and log loss. The means and standard deviations of these metrics are listed in Tables 3, 4, 5, 8, 10, and 11.
