# Supplementary material for: Electronic medical record-based causal network modeling for acute myocardial infarction diagnosis in the emergency department
Source: iScience. 2026 Apr 16;29(5):115742. doi: 10.1016/j.isci.2026.115742 (PMC13146613; doi:10.1016/j.isci.2026.115742)
Supplement: Document S1. Figure S1 and Tables S1–S3 [file mmc1.pdf]

iScience, Volume *29*

Supplemental information

Electronic medical record-based causal network modeling for acute myocardial infarction diagnosis in the emergency department

Bo-Yuan Li, Xue-Qi Li, Yu-Tong Jiang, Xiao-Yang Li, Zhao-Xing Tian, and Rui Kang

SUPPLEMENTAL FIGURE

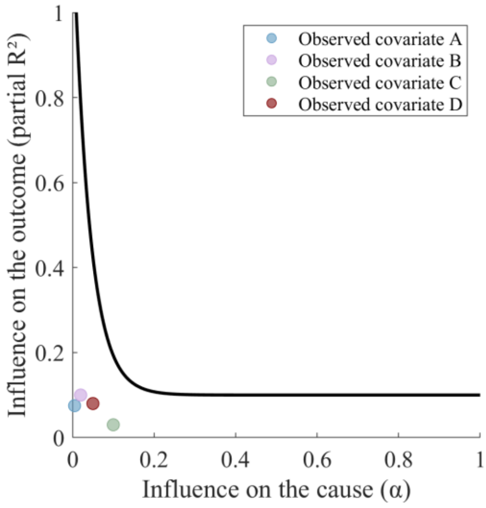

Figure S1. Schematic Austen plot for sensitivity analysis, related to STAR Methods
The dots in an Austen plot presents the effects of observed covariates. If the dots are closer to zero than the black line, it suggests that the validated relationship is robust.

SUPPLEMENTAL TABLES

Table S1. The MEC constructed by FGES with the EMRs collected by authors, related to Figure 3

No.	Cause	Edge	Effect	No.	Cause	Edge	Effect
1	Age	→	D-Dimer	28	DYS	→	HTN
2	Age	→	Nutrition	29	DYS	→	LYM#
3	Alcohol	—	Smoke	30	DYS	→	NEUT
4	AMI	→	ALB	31	DYS	→	TD
5	AMI	→	Chest Pain	32	DYS	→	WBC
6	AMI	→	Dyspnea	33	DYS	→	cTnI
7	AMI	→	HGB	34	HTN	—	CVD
8	AMI	→	Numbness	35	HTN	→	Diabetes
9	AMI	→	STSA	36	HTN	→	CAD
10	AMI	→	TWA	37	HTN	→	SBP
11	CD	→	AMI	38	CAD	→	Diabetes
12	CD	→	CAD	39	CAD	→	PCI
13	CD	→	VHD	40	Nutrition	→	DYS
14	CRI	→	CD	41	Nutrition	→	FIB
15	CRI	→	CS	42	Nutrition	→	INR
16	CRI	→	Cr	43	Nutrition	→	cTnI
17	CRI	→	PCO2	44	Gender	→	Alcohol
18	CRI	→	PH	45	Gender	→	Cr
19	CRI	→	PO2	46	Gender	→	HGB
20	CRI	→	UA	47	Gender	→	Hcy
21	CRI	→	Urea	48	Gender	→	PCI
22	CVD	→	AMI	49	Gender	→	Smoke
23	CVD	→	Numbness	50	Gender	→	TC
24	Diabetes	→	TD	51	Smoke	→	DYS
25	DYS	→	AMI	52	Smoke	→	STSA
26	DYS	→	CD	53	TD	→	ESR
27	DYS	→	Chest Pain	54	TD	→	PR

Table S2. Medical knowledge for the reasons leading to AMI, related to Table 6

Variable<br>EI($X \rightarrow$ AMI)	Mechanism	Reference
DYS	High levels of low-density lipoprotein (LDL) and triglycerides harden arteries and build up the plaques, which clogs arteries and leads to AMI	Ref. [S1,S2]
CD	The cardiac dysfunction can affect blood supply and myocardial metabolism, which may lead to AMI.	Ref. [S3,S4]
CVD	The close hemodynamic coupling for the cardiovascular and the cerebrovascular may affect the blood flow in the heart and lead to AMI.	Ref. [S5]
Nutrition	Malnutrition may lead to the abnormal metabolism of sugar, lipid, and others, which can accelerate the plaque progression and increase the risk of AMI.	Ref. [S6,S7]
Smoke and Alcohol	Smoking and drinking both can promote plaques and the following AMI. Also, their synergistic effects cardiovascular events have been discovered.	Ref. [S8,S9]
CRI	Renal impairment has been found as a potent and independent risk factor for acute coronary syndromes. For instance, it may associate with plaque progression and fibrous cap degradation.	Ref. [S10]
Gender	Females are at lower risk compared with males, due to the protect effect of endogenous female sex hormones.	Ref. [S11,S12]
HTN	Hypertension impairs endothelial cells, elevates the vascular wall stresses, and accelerates the progression of coronary atherosclerosis.	Ref. [S13]
Age	Old people are more likely to suffer AMI, which is related to many factors including increased oxidative stress, inflammation, apoptosis and overall myocardial deterioration.	Ref. [S14,S15]

Table S3. Medical knowledge for the results caused by AMI, related to Table 6

Variable<br>EI(AMI $\rightarrow X$)	Mechanism	Reference
Chest Pain<br>Dyspnea<br>Numbness	These manifestations of AMI reflect that the blood and oxygen supplies to the whole body are insufficient.	Ref. [S16,S17]
STSA<br>TWA	The abnormal ST segment and T wave reflect that the ischemic injury in the cardiovascular affects the transmembrane ion fluxes.	Ref. [S18]
HGB	Due to the inflammation induced by ischemic myocardium, HGB may change.	Ref. [S19,S20]
ALB	ALB is released into the systemic circulation due to the cell necrosis caused by ischemia and hypoxia	Ref. [S21]

SUPPLEMENTAL REFERENCES

[S1] Berliner, J.A., Navab, M., Fogelman, A.M., Frank, J.S., Demer, L.L., Edwards, P.A., Watson, A.D., and Lusis, A.J. (1995). Atherosclerosis: basic mechanisms: oxidation, inflammation, and genetics. Circulation *91*, 2488-2496.

[S2] Wong, N.D. (2014). Epidemiological studies of CHD and the evolution of preventive cardiology. Nature Reviews Cardiology *11*, 276-289.

[S3] Wang, Z.V., Li, D.L., and Hill, J.A. (2014). Heart Failure and Loss of Metabolic Control. Journal of Cardiovascular Pharmacology *63*, 302-313. 10.1097/fjc.0000000000000054.

[S4] Heusch, G. (2022). Coronary blood flow in heart failure: cause, consequence and bystander. Basic Research in Cardiology *117*, 1. 10.1007/s00395-022-00909-8.

[S5] Nakai, M., Iwanaga, Y., Sumita, Y., Wada, S., Hiramatsu, H., Iihara, K., Kohro, T., Komuro, I., Kuroda, T., and Matoba, T. (2022). Associations among cardiovascular and cerebrovascular diseases: Analysis of the nationwide claims-based JROAD-DPC dataset. PloS one *17*, e0264390.

[S6] Czinege, M., Halațiu, V.-B., Nyulas, V., Cojocariu, L.-O., Ion, B., Mașca, V., Țolescu, C., and Benedek, T. (2024). Nutritional Status and Recurrent Major Cardiovascular Events Following Acute Myocardial Infarction—A Follow-Up Study in a Primary Percutaneous Coronary Intervention Center. Nutrients *16*, 1088.

[S7] Gaby, A.R. (2010). Nutritional treatments for acute myocardial infarction. Alternative medicine review : a journal of clinical therapeutic *15*, 113-123.

[S8] Rosoff, D.B., Davey Smith, G., Mehta, N., Clarke, T.-K., and Lohoff, F.W. (2020). Evaluating the relationship between alcohol consumption, tobacco use, and cardiovascular disease: A multivariable Mendelian randomization study. PLOS Medicine *17*, e1003410. 10.1371/journal.pmed.1003410.

[S9] Palatini, P., Mos, L., Saladini, F., Vriz, O., Fania, C., Ermolao, A., Battista, F., Canevari, M., and Rattazzi, M. (2023). Both Moderate and Heavy Alcohol Use Amplify the Adverse Cardiovascular Effects of Smoking in Young Patients with Hypertension. Journal of Clinical Medicine *12*, 2792.

[S10]Pelisek, J., Hahntow, I.N., Eckstein, H.-H., Ockert, S., Reeps, C., Heider, P., Luppa, P.B., and Frank, H. (2011). Impact of chronic kidney disease on carotid plaque vulnerability. Journal of Vascular Surgery *54*, 1643-1649. https://doi.org/10.1016/j.jvs.2011.05.049.

[S11]Pérez-López, F.R., Larrad-Mur, L., Kallen, A., Chedraui, P., and Taylor, H.S. (2010). Gender differences in cardiovascular disease: hormonal and biochemical influences. Reproductive sciences *17*, 511-531.

[S12]Barrett-Connor, E., and Bush, T.L. (1991). Estrogen and coronary heart disease in women. Jama *265*, 1861-1867.

[S13]Konstantinou, K., Tsioufis, C., Koumelli, A., Mantzouranis, M., Kasiakogias, A., Doumas, M., and Tousoulis, D. (2019). Hypertension and patients with acute coronary syndrome: Putting blood pressure levels into perspective. The Journal of Clinical Hypertension *21*, 1135-1143. https://doi.org/10.1111/jch.13622.

[S14]Rodgers, J.L., Jones, J., Bolleddu, S.I., Vanthenapalli, S., Rodgers, L.E., Shah, K., Karia, K., and Panguluri, S.K. (2019). Cardiovascular risks associated with gender and aging. Journal of cardiovascular development and disease *6*, 19.

[S15]Curtis, A.B., Karki, R., Hattoum, A., and Sharma, U.C. (2018). Arrhythmias in patients ⩾ 80 years of age: pathophysiology, management, and outcomes. Journal of the American College of Cardiology *71*, 2041-2057.

[S16]Bozkurt, B., and Mann, D.L. (2003). Shortness of breath. Circulation *108*, e11-e13.

[S17]GORLIN, R. (1965). Pathophysiology of cardiac pain. Circulation *32*, 138-148.

[S18]Klabunde, R.E. (2017). Cardiac electrophysiology: normal and ischemic ionic currents and the ECG. Advances in physiology education *41*, 29-37.

[S19]Padda, J., Khalid, K., Hitawala, G., Batra, N., Pokhriyal, S., Mohan, A., Cooper, A.C., and Jean-Charles, G. (2021). Acute anemia and myocardial infarction. Cureus *13*.

[S20]Yayan, J. (2012). Erythrocyte sedimentation rate as a marker for coronary heart disease. Vascular health and risk management, 219-223.

[S21]Walker, H.K., Hall, W.D., and Hurst, J.W. (1990). Clinical methods: the history, physical, and laboratory examinations.
